# To stress or not to stress: Brain-behavior-immune interaction may weaken or promote the immune response to SARS-CoV-2

**DOI:** 10.1016/j.ynstr.2021.100296

**Published:** 2021-01-27

**Authors:** Eva M.J. Peters, Manfred Schedlowski, Carsten Watzl, Ulrike Gimsa

**Affiliations:** a Psychoneuroimmunology Laboratory, Department of Psychosomatic Medicine and Psychotherapy, Justus-Liebig University Giessen, Giessen and Universitätsmedizin-Charité, Berlin, Germany; b Institute of Medical Psychology and Behavioral Immunobiology, University Hospital Essen, Germany and Department of Clinical Neuroscience, Osher Center for Integrative Medicine, Karolinska Institutet, Stockholm, Sweden; c Department for Immunology, Leibniz Research Centre for Working Environment and Human Factors (IfADo) at TU Dortmund, Dortmund, Germany; d Psychophysiology Unit, Institute of Behavioural Physiology, Leibniz Institute for Farm Animal Biology (FBN), Dummerstorf, Germany

**Keywords:** Stress, Hypothalamic-pituitary-adrenal axis, (Nor)adrenaline, Neuropeptide, COVID-19, Immune suppression, Immune activation, Prevention, Stress reduction

## Abstract

The COVID-19 pandemic continues to strongly affect people with health disadvantages, creating a heavy burden on medical systems and societies worldwide. Research is growing rapidly and recently revealed that stress-related factors such as socio-economic status, may also play a pivotal role. However, stress research investigating the underlying psychoneuroimmune interactions is missing. Here we address the question whether stress-associated neuroendocrine-immune mechanisms can possibly contribute to an increase in SARS-CoV-2 infections and influence the course of COVID-19 disease. Additionally, we discuss that not all forms of stress (e.g. acute versus chronic) are detrimental and that some types of stress could attenuate infection-risk and -progression. The overall aim of this review is to motivate future research efforts to clarify whether psychosocial interventions have the potential to optimize neuroendocrine-immune responses against respiratory viral infections during and beyond the COVID-19 pandemic. The current state of research on different types of stress is summarized in a comprehensive narrative review to promote a psychoneuroimmune understanding of how stress and its mediators cortisol, (nor)adrenaline, neuropeptides and neurotrophins can shape the immune defense against viral diseases. Based on this understanding, we describe how people with high psychosocial stress can be identified, which behaviors and psychosocial interventions may contribute to optimal stress management, and how psychoneuroimmune knowledge can be used to improve adequate care for COVID-19 and other patients with viral infections.

## 1 Introduction

Originating from Wuhan in China, the coronavirus disease COVID-19 reached global spread in just a few months, also and especially in many countries, where infectious diseases were considered to be largely under control until then. Initially, there was hope that the pandemic could be prevented with containment measures, now there is hope that it can be ended with the help of vaccines. However, international experts fear that the waves of infections may only subside within many months, an admission that continues to cause a lot of anxiety and stress in the population as well as in administrative and medical systems worldwide. This dramatic situation calls for the identification of all possible indicators for higher susceptibility to the development of the infection or its complications.

**Fig. 1:**
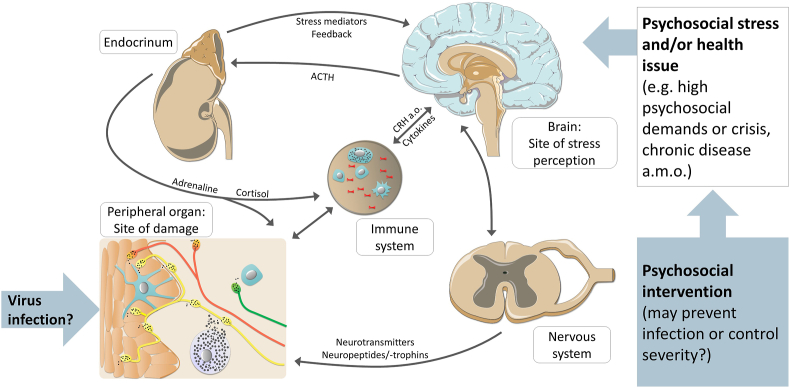
The brain keeps close psychoneuroimmune contact with barrier-forming immune competent tissues such as bronchial, intestinal or cutaneous epithelia. This interaction enables constant interaction between the brain and the periphery, which can affect the control of viral infections of respective organs.

Since socioeconomic issues and various aspects of the Western type lifestyle that are closely associated with psychosocial stress have recently been reported to contribute to COVID-19 (Maroko et al., 2020; Patel et al., 2020), stress could not only be a consequence of the pandemic, but should also be discussed as a potential promotor of respiratory viral infections and associated health deterioration. Research and a discussion of possible stress effects on the immune system's control of respiratory viral infections is therefore timely and urgently needed, to clarify this potential route to escalation and the therapeutic options arising from its understanding. Stress research accumulating over the past decades suggests that differing responses can be expected from more acute versus more chronic psychosocial stress and that neuroendocrine-immune activation provoked by physical and microbial challenges interacts with biomolecular effects of psychosocial stress. Along this line of thought, we hypothesize that certain types of stress may have a direct impact on viral infections by compromising the immune system's effective defense against them (Fig. 1). To follow up on this hypothesis, we compiled a comprehensive narrative review to help shape the development of efficient and successful research efforts on stress-impact and -treatment in COVID-19 and other comparable viral infections.

### 1.1 COVID-19 links with stress-associated health issues and socioeconomic factors

In the past two decades, psychosocial stress has been discussed as one of the causes for the steadily increasing incidence of chronic, non-infectious, non-communicable diseases (NCD) in industrial countries. In this context, pathogenically relevant stress was mostly described as intense, lasting and persistent. It is interesting that people with NCD are now found to be particularly at risk from COVID-19 as it most often affects patients with cardiovascular diseases, chronic obstructive pulmonary diseases, cancer or diabetes mellitus (Li et al., 2020; Yang et al., 2020). Other risk factors, such as obesity and older age as well as nicotine consumption also associate with higher stress levels and psychosocial disadvantages and are common in Western societies (Guan et al., 2020). Moreover, living with social disadvantage associates with both higher stress and worse COVID-19 outcome (Wang et al., 2020). Additionally, a composite measure of socioeconomic deprivation and household income is linked to hospitalizations (Patel et al., 2020), but poverty alone does not seem to explain the reason for this interaction (Maroko et al., 2020). These observations suggest that there is an indirect effect of psychosocial stress on susceptibility to respiratory virus infections through its influence on risk factors.

### 1.2 The first psychoneuroimmune experiments in history demonstrated that psychosocial stress lowers the threshold for viral infections

Hans Selye's original definition of stress in the 1950ies described it as any challenge to the organism that requires an adaptive response. This definition did not describe stress as a potential contributor to disease. It took another decade, to generate the momentum that led to the evolution of an entirely new research field in biomedical research: psychoneuroimmunology. This momentum was generated by the discovery that stress can be a risk- and escalation-factor of disease. Intriguingly, this discovery was brought about by research on respiratory viral infections. In the 1960ies, the first experiments that demonstrated a disease-promoting effect of certain stress paradigms such as sound stress showed that mammals exposed to strong, long-lasting stress were highly susceptible to viruses that enter the body through mucous membranes and cause respiratory infections such as the coxsackie virus (Jensen and Rasmussen, 1963). A number of years later, these experiments were translated to the human level and included rhino- and coronaviruses and stress paradigms such as perceived stress during medical diagnostic procedures (Totman et al., 1977).

Today, we know that stress can have many qualities and therefore can provoke different and sometimes even opposing effects on the neuroendocrine and immune systems as well es mental and physical wellbeing. Accordingly, there are many dichotomizing adjectives for the description of stress such as eustress versus distress, acute versus chronic stress, mild versus severe stress etc., which suggest that stress can have at least two faces. Moreover, both biophysical and psychosocial stress can elicit a neuroendocrine-immune stress response. We here focus on psychosocial stress in the sense of health-promoting versus illness-causing stress and discuss the questions, which neuroendocrine-immune mechanisms are modulated by stress to affect viral respiratory diseases, and whether these mechanisms may constitute intervention targets.

## 2 Lasting psychosocial stress exposure has a negative impact on defence against viral infection

### 2.1 Barrier function of mucosae and skin is affected by psychosocial stress

Stress research over the past decades elucidated a number of mechanisms that could play key roles for the ability of viruses to overcome the immune defense. At the body's interfaces with its environment for example, a quick defense against viruses requires the skin and mucosa of the respiratory tract and intestine to function as a mechanical and biochemical barrier (Le Messurier et al., 2020). Mucosae and skin are dynamic fences, protecting the organism from the entry of foreign agents (“outside-in” barrier) with the help of cell-cell cohesion-enabling tight junctions and surfactant as well as local immune defense peptides such as defensines and lysozyme or mucosal IgA (Basler and Brandner, 2017; Nakatsuji and Gallo, 2012, Wertz and de Szalay, 2020). Cohesion of cells that form the barrier-providing mucosae and epidermis can be disturbed by stress elicited for example by noise to an extent that proliferation and repair is blocked while barrier-forming cells are forced into apoptosis (Peters et al., 2005). When this first line of defense is broken by psychosocial stress elicited for example by academic examinations or chronic social stress and its neuroendocrine mediators (Maarouf et al., 2019), microbes and allergens can enter unhindered and cause the harm they are known for. Moreover, inflammation may spill over from the initial site of infection, and reach for example the brain in form of neuroendocrine mediators and immune cells, and in addition, the blood-brain barrier can be affected (Menard et al., 2017).

### 2.2 Stress mediators that affect barrier function include hormones, neurotransmitters and neuropeptides

Stress-response systems that can compromise barrier function range from the classical hypothalamic-pituitary-adrenal (HPA) axis via the (nor)adrenergic and cholinergic stress axis to neuropeptides and neurotrophins released in response to stress (Blake et al., 2019; Slominski et al., 2012; Suarez et al., 2012). Barrier functions altered directly by stress include the decrease of epidermal hydration and increased transepidermal water loss due to the decrease of epidermal lipids and structural adhesion proteins, both of which allows viruses to trespass the barrier more easily. In addition, innate immune cells that reside in the epidermis-underlying dermis such as the mast cells can be activated by stress to release histamine and inflammatory cytokines that further compromise barrier function (Kempuraj et al., 2020; Kritas et al., 2020).

A recent review summarized 21 studies showing the negative impact of the HPA-axis activation on skin-barrier function (Maarouf et al., 2019). Perceived stress was also shown to increase mucosal cortisol production by increasing beta-hydroxysteroid dehydrogenase type I levels, a process that was reversible by treating anxiety with serotonin-reuptake inhibitors (Choe et al., 2018). Interview and marital dissolution stress reportedly decreased skin-barrier recovery after tape stripping up to 24 h after the tape strip in women and at the same time increased noradrenaline in addition to cortisol (Altemus et al., 2001; Muizzuddin et al., 2003), while essential proteins for endothelial barrier function such as tight-junction molecules are down regulated by noradrenaline (Ittner et al., 2020; Martin et al., 2017). In addition, the cholinergic system of the skin and mucous membranes seems to play a special role in the stability of the barrier, as chronic, long-term psychosocial stress can compromise the skin's barrier function by modulating nicotine receptors on epithelial cells (Curtis et al., 2012). Psychosocial stress was also shown to disrupt the function of the intestinal barrier (Barreau et al., 2007; Moussaoui et al., 2017) and in this context the agonists of the beta-adrenergic receptor and the neuropeptides corticotropin-releasing hormone (CRH) and substance P (SP) were found to enhance barrier permeability (Keita et al., 2010; Sorribas et al., 2020), while mast cell stabilizers and neutralization of the neurotrophin nerve growth factor (NGF) were able to improve barrier function (Barreau et al., 2007; Kritas et al., 2020).

### 2.3 Angiotensin-converting enzyme 2 can tilt the balance between pro- and anti-inflammatory immune responses to stress

It is interesting in the context of SARS-CoV-2 that the virus uses angiotensin-converting enzyme 2 (ACE2), a membrane-bound enzyme that is localized on type II pneumocytes, to overcome the barrier and bind to host cells in the alveoli. These cells produce the surfactant and are crucial for the barrier function of the alveoli (Verdecchia et al., 2020). Additional barrier-relevant cells that express ACE2 are endothelial cells in the heart, kidney and intestines (Varga et al., 2020). When the virus has entered ACE2-positive cells, it down-regulates the expression of the enzyme (Zhang et al., 2020a). ACE2 normally binds angiotensin II, a molecule best known for its role in blood pressure regulation but recently also acknowledged as a key player in the conventional renin-angiotensin system (RAS) that promotes inflammation, oxidative stress and apoptosis (Sharma et al., 2019). Since the enzyme converts the pro-inflammatory angiotensin II into the anti-inflammatory angiotensin 1-7 after its binding to angiotensin receptors, the downregulation of ACE2 exerts a pro-inflammatory effect. Accordingly, an increased production of angiotensin 1–7 has a protective effect on the endothelial barriers of the lungs, kidneys, heart and intestines (Chen et al., 2018; Fang et al., 2019; Sharma et al., 2019), and protects patients with viral infection of the respiratory tract from deleterious inflammation. This observation is highly relevant in the context of stress because ACE2 plays a role in neuro-immune interaction and angiotensin 1–7 has a protective effect also on the heart and on the gastrointestinal barrier under psychosocial stress (Brzozowski et al., 2012; Calvillo et al., 2019). In addition, at the intestinal barrier of mice, the pro-inflammatory effect of a two-week intermittent restraint stress paradigm can be promoted by blocking the angiotensin receptors Ia and II (Yisireyili et al., 2018). At the same time, angiotensin-receptor blockade increases the expression of angiotensin 1-7 in cardiac tissue and brain of stressed experimental animals, thus preventing microvascular fibrosis and stress-induced increase in heart rate (Firoozmand et al., 2018; Fontes et al., 2016). These processes can be relevant during early infection as well as with respect to the long-term outcome and tissue damage caused by lack of angiotensin 1-7.

### 2.4 Following barrier breach, stress mediators can hamper an orchestrated immune response against viruses

Excellent reviews have summarized how sensitive the process of host defense is to psychosocial stress and stress reduction (Dhabhar, 2014; Marsland et al., 2017; Schakel et al., 2019). Some describe the complex interaction between the immune system, the functional tissue of mucosae and the environment as the mucosally associated lymphoid tissue and ascribe it a potent role in the control of respiratory viral infections (Gallo et al., 2020). The immune response within such tissues can be regulated by stress mediators. Prolonged stress exposure for example in a social instability stress paradigm, increased noradrenergic innervation of lymphoid organs, which reduced the activity of the innate type I interferon system (Capitanio and Cole, 2015). This system is the earlierst immune defense against viruses and is responsible for an effective removal of virus-infected cells (Chan et al., 2020; Wan et al., 2020) through support of natural killer (NK) cell activation and promotion of viral antigen presentation to T lymphocytes (Durbin et al., 2013). The immunopharmacotherapy of viral diseases with type I interferon is a good example for a targeted immune intervention that can overcome stress-induced suppression of this defense system and in COVID-19 treatment appears to be especially effective if applied in the early stages of a SARS-CoV-2 infection, as the virus itself suppresses type I-interferon production (Khan etal., 2020; Kikkert, 2020).

**Fig. 2:**
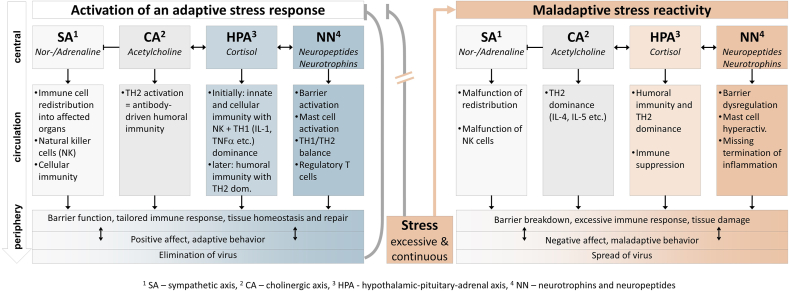
Adaptive interaction of neuroendocrine-immune processes (left panel) transforms into maladaptive interactions in the presence of chronic stress (right panel).

The HPA axis appears to play another important role in this context via its end product cortisol (Fig. 2). However, cortisol seems to be a double-edged sword. On the one hand, stress-increased endogeneous cortisol production is associated with higher susceptibility to virus infections of the respiratory tract (Janicki-Deverts et al., 2016). This can be explained by the mode of action of cortisol, which suppresses NK cell activity (Callewaert et al., 1991) and represses the transcription of proinflammatory cytokines via a direct interaction of the glucocorticoid receptor with the transcription factor NFκB (Newton, 2000). In addition, prolonged HPA activation or treatment with high doses of cortisol derivatives can hamper efficient host defense by downregulating innate and cellular immune defense mechanisms (Theoharides and Conti, 2020), a process associated with CD8^+^ T-cell senesence and higher viral load in inflamaging (Bauer et al., 2015). Moreover, negative feedback and down-regulation of glucocorticoid receptors in response to chronically increased cortisol production can lead to glucocorticoid resistance, which in turn promotes an increased and prolonged production of cytokines in the course of viral inflammation (Cohen et al., 2012; Janicki-Deverts et al., 2016). This could promote the cytokine storm reported in critically ill COVID-19 patients. Finally, chronic psychosocial stress, possibly via cortisol, could have a negative impact on the development of antibodies against viruses (Miller et al., 2004). Indicators of a negative impact of cortisol on defense mechanisms against respiratory viruses are thus abundant. On the other hand, cortisol is long known to suppress hyperinflammatory responses in infectious disease when given in high doses (Jung et al., 2007; Zhang et al., 2020b). COVID-19 patients suffering from a cytokine storm therefore respond well to treatment with its derivatives (Ledford, 2020). However, this requires very high doses of exogeneous steroids to overcome glucocorticoid resistance.

### 2.5 Cytokines are intermediaries between neuroendocrine stress responses and immunogenic virus-control

Often stress studies addressing the biomolecular impact of stress on health report primarily cytokines as an instructive outcome that can indicate the type of immune response favorably produced in a given stress context as well as the health-altering impact that this immune response may have. The more acute the respective psychosocial stress is, the stronger is the activation of the innate immune response and the higher is the production of interferons and other pro-inflammatory cytokines such as interleukin (IL)-1, IL-6 or tumor necrosis factor alpha (TNFα) (Dhabhar, 2018; McInnis et al., 2015), which show a dramatic but transient increase within the first 2 h of a psychosocial stress encounter (Marsland et al., 2017). This boosts for example vaccine responses and post-operative recovery but can also contribute to the fatal effects of a cytokine storm (Kempuraj et al., 2020). Moreover, psychological states that are associated with repeatedly high stress states such as depression or PTSD are also associated with a chronic low-grade increase of these cytokines (Speer et al., 2018; Troubat et al., 2021). Vice versa, cytokines produced in response to an inflammatory challenge can affect the HPA, closing a vicious cycle (Straub et al., 2011). However, the more chronic the stress, the more the immune response follows the necessity to optimize energy expenditure (Straub et al., 2011) and shifts to the more selective adaptive immune response, T-helper cell type 1 (TH1) and the T-helper cell type 2 (TH2) responses, and the more cytokines such as interferon-gamma (IFNγ), IL-4, IL-5 and IL-10 are produced (Elenkov, 2004). These cytokines are often labeled as anti-inflammatory as they terminate the initial pro-inflammatory response (Dhabhar, 2014). However, in allergy and cancer this immune response can have detrimental effects (Peters et al., 2012) and of further interest in this context, cytokine production maybe shifted to this response by increased cortisol in the context of a respiratory viral infection (Pinto et al., 2006). Only if stress lasts very long the production of cytokines is compromised and a general immune suppression can be observed.

### 2.6 Oxidative stress generated by psychosocial stress, an unhealthy life-style and inflammation can worsen negative stress outcomes

Oxidative stress results both from the environment (e.g. UV light, heavy metals, cigarette smoke) and from endogenous processes, such as the enzymatic production of reactive oxygen species (ROS) by mitochondria or by neutrophils activated by danger-associated molecular patterns during inflammation (Finnell and Wood, 2018; Marx et al., 2017; Schiavone et al., 2013). A direct link between glucocorticoids and oxidative stress has been demonstrated in mice exposed to psychosocial stress (Lee et al., 2009). This is interesting as among many other potential mechanisms, oxidative stress is discussed as the cause of more severe COVID-19 infections in elderly people with reduced antioxidant capacity as well as the cause of the very mild symptoms in children with highly potent antioxidant systems (Cecchini and Cecchini, 2020; Iddir et al., 2020; Keles, 2020).

### 2.7 Obese people are particularly at risk for an imbalanced interaction of psychosocial stress, inflammation and immune responses

Individuals with an obese body mass index (BMI) respond to repeated psychosocial stress with an overproduction of IL-6, a marker cytokine of low-grade inflammation that strongly associates with negative health outcomes (McInnis et al., 2014). Elevated levels of IL-6 among other factors of innate immune hyperactivation are considered responsible for a more severe course of disease of COVID-19 patients (Vardhana and Wolchok, 2020). Elevated levels of IL-6 counteract NK cell and CD8^+^ T cell responses and thereby reduce the capacity of obese people to mount an effective immune response to viral challenge, which renders them more susceptible to infection (Hou et al., 2014). In addition, IL-6 and chronic psychosocial stress promote the accumulation of immature myeloid-derived suppressor cells (Mohammadpour et al., 2019). These cells are elevated in obese people and suppress the antiviral activity of NK cells and CD8^+^ T cells (Bao et al., 2015; O'Connor et al., 2017). These mechanisms are highly relevant in the case of SARS-CoV-2 infections. A recent study showed that both obesity and a lower number of CD8^+^ T cells predict a more severe course of the disease and that CD8^+^ T cell counts are negatively correlated with IL-6 levels in the blood of COVID-19 patients (Jiang et al., 2020; Urra et al., 2020). Furthermore, obesity is often associated with an unhealthy diet, while a healthy diet promotes a healthy gut microbiome, which in turn stabilizes the intestinal barrier (Selhub et al., 2014), regulates serotonin metabolism (O'Mahony et al., 2015) and interacts with the HPA axis (Tetel et al., 2018). It should be noted here that obesity should be better divided into metabolically abnormal and metabolically healthy obesity, the latter characterized by the absence of diabetes but also by cardiorespiratory fitness (Ortega et al., 2015). It is conceivable that metabolically healthy obese individuals are at lower risk for increased COVID-19 severity because they have no immune-system impairment compared with obese individuals with diabetes (Richard et al., 2017).

## 3 Which stress-reducing interventions may be suitable to prevent or improve viral respiratory diseases?

**Fig. 3:**
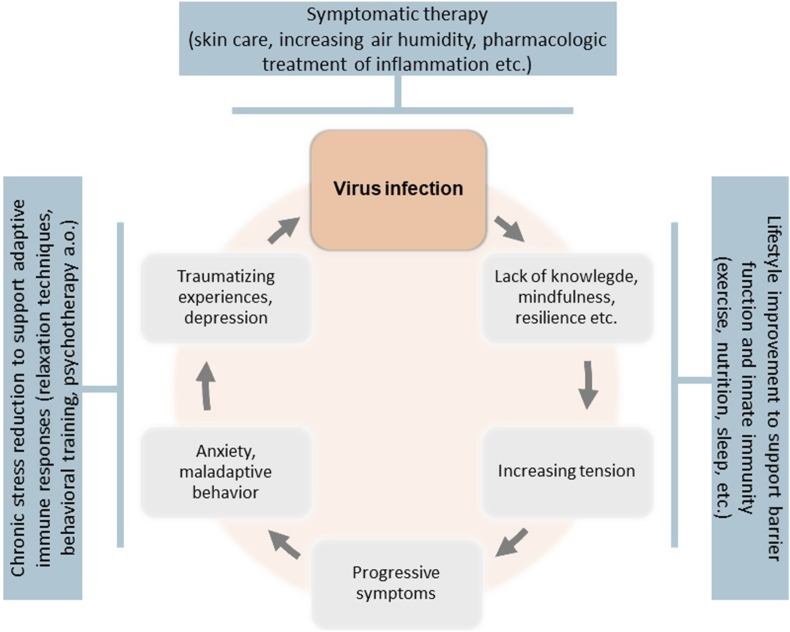
Psychological factors that exacerbate virus infection in a vicious circle. Psychosocial interventions may interfere with the exacerbation and thereby prevent or improve viral infection.

Interestingly, many of the Western type lifestyle aspects that generate persistant and high levels of health-compromising stress can be addressed directly or indirectly through changes of behavior and improvement of health literacy. This includes for example measures to improve physical activity, sleep, relaxation and eating habits (Della Giustina et al., 2020; Zhu et al., 2016) (Fig. 3). In addition, a plethora of psychosocial measures exist that have the capacity to reduce stress-generating mental health issues. These range from relaxation exercises via schooling programs for health behaviors to behavioral and psychodynamic therapy, all of which may help to control disease-promoting stress responses (Chan et al., 2020). With a look at the health-deteriorating effects of chronic psychosocial stress, measures that reduce chronic stress seem to have a high potential to also reduce susceptibility to virus-related respiratory infections and to favorably influence the course of respective disease.

Following an integrated bio-psycho-social concept, measures to stabilize barrier functions may hence combine external treatment with moisturizing and anti-inflammatory ointments, termination of chronic nicotine consumption, stress reduction training and psychotherapy into an integrated multi-modal treatment approach (Heratizadeh et al., 2017) (Fig. 3). This may help to improve the prevention of viral infection of the respiratory system and its complications. However, evidence for the effectiveness of such integrative treatment approaches in COVID-19 is largely missing to date and at present recommendations have to rely on analogies as described below.

However, against the background of psychoneuroimmune mechanisms that can play a role in the interaction between psychosocial stress and viral infections, both the promotion of acute stress responses as well as the reduction of chronic stress effects appear to offer promising avenues for the prevention and improvement of infectious disease. Short-acting psychosocial stress alternating with recovery phases may possibly be used to train the innate immune defense and promote an anti-viral immune response, while chronic psychosocial stress can be addressed by stress prevention- and stress reduction-strategies (McInnis et al., 2015). This health-deteriorating stress may be reduced by a plethora of interventions and treatments that can range from placing the patient in a supportive psychosocial environment, via increasing stress-regulating behaviors and -coping capacities, to measures strengthening stress resilience and resolving mental health issues. This is important, since development of symptoms of anxiety and post-traumatic stress disorder have been observed in patients with COVID-19 (Bo et al., 2020) as well as in the general population exposed to the COVID-19 pandemic (Jungmann and Witthoft, 2020). At the same time, suggestive evidence exists that relaxation techniques, behavioral therapy, psychodynamic procedures and other psychotherapeutic approaches may all be effectively reducing the health deteriorating effects generated for example by life adversities or smoldering conflicts such as posttraumatic stress, anxiety or depression. Effective coping capacities and high resilience can be achieved by respective interventions and counteract chronic psychosocial stress (Finnell and Wood, 2018; Zozulya et al., 2008). A selection of these measures are discussed in detail below.

### 3.1 Acute psychosocial stress can have positive effects on the defense against viruses

Paradoxically, while chronic psychosocial stress is considered to promote viral illness as described above, acute psychosocial stress can stimulate the immune system to protect from microbial intruders by stabilizing barriers in lung, gut and skin and by activation of rapidly acting innate as well as cellular adaptive TH1 immune responses. Correspondingly, acute infection shares many features with an acute response to psychosocial stress, both of which increase the levels of IL-1β, IL-6 and TNFα (Marsland et al., 2017); cytokines, which are also known to boost the response to vaccine in short-term stress paradigms such as single restraint-stress exposure in mice (Dhabhar and Viswanathan, 2005).

This acute innate and TH1 immune response interacts positively with an activated adrenergic stress axis and rapid while transient HPA activation as well as with neuropeptidergic and neurotrophinergic stress mediators (Dhabhar, 2018; Kolmus et al., 2014; Peters et al., 2005; Taylor et al., 2012). Endogenously produced (nor)adrenaline and cortisol for example initially promote host defense mechanisms as they transiently increase IL-1β, TNFα and NK-cell activity. Increased cortisol also leads to higher neutrophil activity in the oral cavity (Gomaa et al., 2020). Likewise, acute increases of adrenaline induced for example by exercise stress can mobilize innate immune-defense cells such as NK cells, non-classical monocytes and differentiated subsets of CD8^+^ T-cells (Graff et al., 2018). Acute, short-term psychosocial stress can also mobilize endogenous factors such as secreted Ly-6/uPAR related protein-1 (SLURP1) that neutralize the infection-promoting effects of nicotine on its receptors by stabilizing barrier function and promoting T-cell activation (Campbell et al., 2019; Peters et al., 2014; Razani-Boroujerdi et al., 2004; Tjiu et al., 2011). Finally, acute psychosocial stress leads to the rapid but transient release of neuropeptides such as SP from peripheral nerve endings in mucosae and skin, and this may contribute to first-line anti-viral defense by short-term upregulation of toll-like receptors (TLRs) (Larsson et al., 2018; Yarmohammadi et al., 2021).

### 3.2 Life-style factors that reduce psychosocial stress and health-compromising neuroendocrine-immune stress responses

#### 3.2.1 Sleep hygiene

Achieving sufficient and regular undisturbed sleep can counteract the development of harmful stress biomolecular responses and secures antiviral immune defense. In fact, a number of studies show that sleep deprivation decreases NK cell activity (Besedovsky et al., 2019). Similarly, lack of sleep is associated with an increased susceptibility to respiratory infections (Prather and Leung, 2016). One reason for this could be that mild chronic inflammation is associated with sleep disorders (Besedovsky et al., 2019).

#### 3.2.2 Regular endurance sports

This recommendation includes e.g. gymnastics, walking, cycling, gardening a.o., if possible outdoors, all of which can improve neuroendocrine-immune stress responses (Sandifer and Walker, 2018; Weyh et al., 2020). It is important to ensure, however, that the exercise is moderately aerobic as this has an activating effect on innate immune defense and is therefore protective against respiratory diseases. Moderate sportive activity increases for example NK-cell activity (Barra et al., 2017) and upregulates the immune-protective angiotensin 1-7 (Frantz et al., 2018). In contrast, more intense sportive activity can have suppressive effects on innate immunity (Martin et al., 2009).

#### 3.2.3 Nutritional interventions for mental health

This relatively new field in psychosocial medicine requires a more indepth assessment as the role of nutrition in mental illness such as depression and anxiety is presently widely studied but recommendations are complex. For a start, there is consistent epidemiological evidence of links between diet quality and mental health, suggesting that healthy food (i.e. rich in vegetables, fruit, whole grains, seeds, nuts, fish) is inversely associated with the risk of depression, while an unhealthy diet (i.e. processed foods high in fat and sugar) is associated with depression and anxiety (Adan et al., 2019; Chang and Su, 2020; Jacka, 2017; Marx et al., 2017; Selhub et al., 2014). The psychoneuroimmune mechanisms by which a healthy diet influences the brain are manifold, e.g. modulation of neurotransmitters and neurotrophins, reduction of inflammation, and reduction of oxidative stress (Marx et al., 2017; Wang et al., 2018; Watzl et al., 2005).

A change in diet to healthy foods has clinical benefits in the treatment of mental illness (Adan et al., 2019; Jacka et al., 2017). Nutraceuticals (food supplements) such as omega-3 polyunsaturated fatty acids (ω−3-PUFAs), N-acetylcysteine, S-adenosylmethionine, B- and D-vitamins, pre- and probiotics promise a more selective intervention against known deficiencies (Fernandez et al., 2019). Nutritional supplementation with ω-3-PUFAs blunted effects of psychosocial stress and reduced anxiety in healthy volunteers (Delarue et al., 2003; Kiecolt-Glaser et al., 2011) and may even be therapeutic in major depression (Bazinet and Laye, 2014; Bazinet et al., 2020). Interestingly, the same vitamins and trace elements that are necessary for mental health (Kris-Etherton at al., 2020; Wang et al., 2018) are also essential for the proper functioning of the immune system (Wintergerst et al., 2007). Selenium deficiency for example not only weakens the immune response against viruses, but may even increase virulence of viruses by causing mutations in the viral genome, probably caused by oxidative stress in the host cells (Bazinet et al., 2020; Beck et al., 2004). Moreover, probiotics have been shown to influence the HPA axis (Sudo et al., 2004), modulate the neuroendocrine stress response (Dinan and Cryan, 2012) and shape the immune system (Hooper et al., 2012). Prebiotics that feed the “right” intestinal microbiota can have anxiolytic effects and reverse the impact of chronic psychosocial stress. This was demonstrated in mice, which were fed a combination of fructo-oligosaccharides and galacto-oligosaccharides over a period of 6 weeks while subjected to chronic unpredictable social stress. Behavioral tests were performed during the last 3 weeks of the study. Chronically stressed mice that were fed these prebiotics had significantly lower levels of plasma corticosterone and reduced production of IL-6 and TNFα in spleen cells following mitogenic stimulation. In addition, they showed less depressive behavior in the forced swim test and the tail suspension test and reduced anxiety in the elevated plus maze compared with stressed mice on standard chow (Burokas et al., 2017). In summary, the antiviral defense of the immune system is supported by optimal nutrition (Calder et al., 2020) and the nutritional status is a promising target for intervention against SARS-CoV-2 infections, as it integrates both mental health and immune defense.

### 3.3 Resilience factors that promote adaptive neuroendocrine-immune responses to psychosocial stress

#### 3.3.1 Humor

Humor has proven to be a potent resilience factor. The saying “laughter is the best medicine” has been verified by numerous studies, and in fact it seems that activities that elevate the mood and lead to laughter such as watching funny movies can exert positive effects on both mental and physical illnesses (Nasr, 2013). Laughter activates the sympathetic nervous system, increases the heart and respiratory rate and oxygen consumption (Fry and Savin, 1988) and a number of studies found that laughter increases NK-cell activity (Bennett et al., 2003). This increase in NK-cell activity can be a direct effect of humorous stress reduction, but also an indirect one by lowering the cortisol level (Berk et al., 1989).

#### 3.3.2 Social inclusion

Social integration may be an effective momentum in the fight of the immune system against viral respiratory diseases. It was shown that high social integration can reduce susceptibility to viral respiratory diseases (Cohen et al., 2003). This may be explained by the finding that social support can buffer negative long-term stress effects (Janicki Deverts et al., 2017), a relationship recently confirmed in COVID-19 patient care providers (Xiao et al., 2020). The reduction of tension that social deficits cause therefore seems to be a worthwhile goal, especially in times of forced social isolation, social distancing and quarantine (Brooks et al., 2020).

### 3.4 Relaxation techniques to control deregulated neuroendocrine-immune responses to psychosocial stress

The term relaxation techniques summarizes procedures that allow the person practizing relaxation techniques to attain a state of physical as well as mental calmness. A selection of these techniques and their neuroendocrine-immune effects potentially relevant to respiratory viral infections are discussed below.

#### 3.4.1 Yoga

Originally developed in India, Yoga has evolved into a relaxation technique that integrates mind and body through defined physical exercises combined with breathing exercises and elements of meditation with the aim to attain a vitalized state of inner calm. Especially Yoga that does not exhaust was shown to reduce evening cortisol as well as waking cortisol, systolic blood pressure, heart rate, heart-rate variability, fasting blood glucose, cholesterol and low density lipoprotein in a meta-analysis comparing it to active control paradigms (Pascoe et al., 2017). Heart-rate variability thereby measures the natural variation in time between two consecutive heartbeats, which is thought to be a measure for the flexibility of the autonomous nervous system to adapt to stress (Perna et al., 2020).

#### 3.4.2 Mindfulness-based stress reduction (MBSR)

Mindfulness is a concept that trains a perception of oneself and one's surroundings that is deliberate and non-judgmental and stays in the present moment. This increases the ability to perceive bodily sensations and mood unbothered from stress (Barrett et al., 2012; Creswell et al., 2009; Hayney et al., 2014). MBSR can reduce psychosocial stress, anxiety and depression (Dundas et al., 2016; Witek Janusek et al., 2019), restore HPA activity (Manigault et al., 2019), reduce adrenergic activation and neurogenic inflammation (Carlson et al., 2007; Rosenkranz et al., 2012), decrease CRP and pro-inflammatory cytokines (Creswell et al., 2012; Witek Janusek et al., 2019) and increase NK-cell activity (Fang et al., 2010; Witek Janusek et al., 2019) as well as CD4^+^ T cell counts (Creswell et al., 2009) with sufficient practice in the procedure (Moraes et al., 2018; Reive, 2019). Also, there is suggestive evidence that it protects from respiratory virus infection (Barrett et al., 2018). However, it failed to improve vaccine responses to influenza (Hayney et al., 2014).

#### 3.4.3 Progressive muscle relaxation (PMR)

This technique is based on the simple exercise of first briefly tensing and then deliberately relaxing defined groups of muscles. PMR was shown to be able to control viral infections almost 40 years ago (VanderPlate and Kerrick, 1985) and was shown to associate with adrenergic activation and increased IgA levels around the same time (Jasnoski and Kugler, 1987). In isolated COVID-19 patients, it reduces anxiety and improves sleep (Liu et al., 2020), its effect on immune function in these patients however has yet to be shown and previous studies on chronic inflammatory disease failed to demonstrate immunological effects (Bae et al., 2012). Likewise, PMR was shown in some studies to increase (Beddig et al., 2020) and in others to decrease cortisol (Jasnoski and Kugler, 1987; Pawlow and Jones, 2005).

#### 3.4.4 Autogenic training

This relaxation technique involves passive concentration and repetitive self-suggestions and visualisations of certain bodily perceptions (e.g. the warmth in your hands) and is known to reduce hyper-inflammatory responses in the context of allergic diseases for quite some time (Chida et al., 2007). It is also known to decrease anxiety and depression (Manzoni et al., 2008; Ramirez-Garcia et al., 2019), and increase heart-rate variability (Seo and Kim, 2019) in patients with diseases such as breast cancer or HIV. However, its neuroendocrine-immune effects are scarcely studied.

#### 3.4.5 Closed-loop, allostatic, acoustic stimulation neurotechnology

This acoustic stimulation neurotechnology produces real-time translation of dominant brain frequencies into audible tones of variable pitch and timing to support the auto-calibration of neural oscillations. This less known intervention was shown to achieve an improvement in the ratio of angiotensin II to angiotensin 1-7 in the context of post-traumatic stress disorder (Tegeler et al., 2017).

#### 3.4.6 Forest bathing

Spending time in nature is an easy way for many people to calm down and relax. Forest bathing (shinrin-yoku) is a traditional Japanese practice combining walking in a forest, watching it and breathing the pleasant aromas as a kind of meditation. It has gained scientific interest because of its stress-reducing effects. Indeed, a systematic review and meta-analysis has confirmed its cortisol-decreasing potential (Antonelli et al., 2019). Also, forest bathing may reduce pulse rate, increase vigor and reduce fatigue, anxiety and depression (Li et al., 2016). A pilot study observed an increase in NK-cell activity (Tsao et al., 2018). Part of the effect can be attributed to volatile organic compounds that are inhaled (Antonelli et al., 2020). Even though placebo effects cannot be ruled out, forest bathing is still recommended for many reasons.

### 3.5 Psychotherapy for long-term psychosocial stress reduction and recalibration of health promoting neuroendocrine-immune responses

Sustained changes in the neuroendocrine-immune responsiveness to psychosocial stress exist in people who were neglected or traumatized during early development or experienced critical conflicts in their youth and could not resolve them. In later life, they are characterized by a chronically altered stress responsiveness and show behaviors or make experiences that maintain chronic psychosocial stress (Bauer et al., 2010; Groer et al., 2015). These patients have been underprovided during the COVID-19 pandemic (Probst et al., 2020). From a therapeutic point of view, it is vital to address these early developmental deficits and lasting conflicts because if adequately addressed, affected patients can be expected to show improved neuroendocrine-immune stress responses with consequences for viral disease susceptibility and complications. Appropriate therapeutic procedures are provided for example by behavioral therapies such as Cognitive Behavioral Analysis System of Psychotherapy (CBASP) or they are summarized under the term psychodynamic therapy, which includes classic psychoanalysis as well as modern manualized short-term psychodynamic therapies (Ewin, 2011; Pitman and Knauss, 2020; Serbanescu et al., 2020) as discussed in more detail below. However, research into the realm of neuroendocrine-immune mechanisms of virus control in mentally ill patients has yet to be performed.

#### 3.5.1 Behavioral therapy

Behavioral therapy is a psychotherapeutic approach that promises a more long-term psychosocial stress, anxiety and depression reduction (McIntosh et al., 2019). In addition to the restoration of normal HPA and adrenergic activity as well as other neuroendocrine effects that can be achieved (Heckenberg et al., 2018; Manigault et al., 2019; Moraes et al., 2018), it reduces CRP and pro-inflammatory cytokines such as TNFα and IL-6 (Heckenberg et al., 2018; Moreira et al., 2015) and improves leukocyte transcriptional profiles and response to virus vaccination (Antoni et al., 2016; Heckenberg et al., 2018).

#### 3.5.2 Psychodynamic therapy

The psychodynamic approach addresses psychological defense mechanisms and the disquieting feelings and basic needs that often drive our more negative interactions with others. The present discussion of if, when, where and how to wear masks and what the necessary distance is to prevent virus spread gives a vivid example of the psychosocial stress generated by psychodynamic processes in the context of the COVID-19 pandemic. To address psychodynamic aspects of the pandemic and viral disease can therefore be instructive and stress-reducing (Kent and Blumenfield, 2011; Marcinko et al., 2020; Rogers, 1989). If psychodynamic therapy has the capacity to improve neuroendocrine-immune virus control, however, is open to debate.

## 4 How can we identify highly stressed individuals in the context of infection risks?

In the context of an impending virus-related respiratory disease, the question arises, how individuals who are premorbidly affected by chronic psychosocial stress can be identified in order to provide best care for them as early as necessary and possible. This is especially important because timely intervention appears to be a key factor in reducing mortality (Zhang et al., 2020c). One option seems to be the use of health questionnaires such as the Short Form Health Survey (SF-12), because a positive self-assessment of the premorbid state of health was shown to predict positive outcome of viral respiratory disease in various studies. In turn, low quality of life may indicate a premorbid immune constellation that favors a negative course of the disease (Cohen et al., 2015). Self-reported quality of life could therefore be used to identify individuals who need special protection and early intervention. Other possible indicators for high psychosocial stress include symptoms of depression, anxiety symptoms and the perception of high tension, which can be assessed by validated self-assessment questionnaires such as the Patient Health Questionnaire (PHQ), the State and Trait Anxiety Index (STAI) or the Perceived Stress Questionnaire (PSQ). If experimental and clinical studies of COVID-19 would include assessment of quality of life and other indicators of chronic psychosocial stress, it would become possible to learn, whether this identifies a subpopulation of high-risk individuals and if these instruments have predictive qualities for the development of the infection and an unfavorable course of the disease. In addition, if combined with biological data, their employment could help to clarify, which neuroendocrine-immune mechanisms are behind a higher susceptibility to infections with SARS-CoV-2 in high-risk population sub-groups.

## 5 Conclusion

Stress is generally regarded as pathogenic, with abundant data supporting a detrimental neuroendocrine-immune effect of chronic psychosocial stress on viral infections. Reduction of chronic psychosocial stress can therefore have beneficial effects and potentially prevent infections or contribute to a milder course of viral respiratory diseases. However, with regard to the appraisal of stress in connection with infection-control measures, the pendulum is now swinging in the opposite direction and reports are accumulating that acute challenges to neuroendocrine activation, i.e. short intense stress response activating events, can improve the immune defense against viruses. The antidote for a virus-favoring immune constellation therefore seems to be a well-trained neuroendocrine-immune stress response, which can be generated by a balanced degree of activation alternating with relaxation, and that shapes an immune response that is optimally equipped for the challenges imposed by new infectious agents.

## CRediT authorship contribution statement

**Eva M.J. Peters:** Conceptualization, Writing - original draftWriting – original draft, Writing - review & editingWriting – review & editing, the conception and design of the review, drafting the article, editing and revision, critically revising it for important intellectual content, final approval of the version. **Manfred Schedlowski:** Conceptualization, Writing - original draftWriting – original draft, Writing - review & editingWriting – review & editing, the conception and design of the review, drafting the article, editing and revision, critically revising it for important intellectual content, final approval of the version. **Carsten Watzl:** Conceptualization, Writing - original draftWriting – original draft, Writing - review & editingWriting – review & editing, the conception and design of the review, drafting the article, editing and revision, critically revising it for important intellectual content, final approval of the version. **Ulrike Gimsa:** Conceptualization, Writing - original draftWriting – original draft, Writing - review & editingWriting – review & editing, the conception and design of the review, drafting the article, editing and revision, critically revising it for important intellectual content, final approval of the version.

## Declaration of competing interest

The authors declare no conflict of interest.
